# Smoking: additional burden on aging and death

**DOI:** 10.1186/s41021-016-0029-9

**Published:** 2016-01-22

**Authors:** Masahiko Watanabe

**Affiliations:** School of Pharmacy, Shujitsu University, 1-6-1 Nishigawara, Okayama, 703-8516 Japan

**Keywords:** Cigarette smoking, Smoking cessation, Dose–response relationship, Aging, Cancer risk

## Abstract

Tobacco smoking is a major cause of lung cancer. It has been suggested that there is an approximately linear dose–response relationship between the number of cigarettes smoked per day and clinical outcome such as lung cancer mortality. It has also been proposed that there is a greater increase in mortality at high doses when the dose is represented by the duration of the smoking habit rather than the number of cigarettes. The multistep carcinogenesis theory indicates that a greater increase in mortality rate at high doses is possible, as is the case between aging and cancer, even though each dose–response relationship between a carcinogenic factor and a carcinogenic step forward is linear. The high incidence of lung cancer after long-term smoking and the decreased relative risk after smoking cessation suggests a similarity between the effects of smoking and aging. Prediction of lung cancer risk in former smokers by simple integration of smoking effects with aging demonstrated a good correlation with that estimated from the relative risk of the period of smoking cessation. In contrast to the smoking period, there appears to be a linear relationship between smoking strength and cancer risk. This might arise if the dose–response relationship between smoking strength and each carcinogenic step is less than linear, or the effects become saturated with a large dose of daily smoking. Such a dose–response relationship could lead to relatively large clinical effects, such as cardiovascular mortality, by low-dose tobacco smoke exposure, e.g., second-hand smoking. Consideration of the dose–response of each effect is important to evaluate the risk arising from each carcinogenic factor.

## Introduction – smoking as a human carcinogen

Smoking causes a variety of diseases including cancer. In 1981, Doll and Peto estimated that 30 % of all cancer deaths were attributable to tobacco smoking [1]. This estimation is basically similar in Japan, as the population attributable fractions of cancer mortality related to tobacco smoking in Japanese men and women in 2005 have been estimated to be 34.4 % and 6.2 %, respectively [2]. Tobacco smoke contains many kinds of carcinogens. The International Agency for Research on Cancer evaluated and classified 16 compounds present in tobacco smoke as Group 1 - carcinogenic to humans [3]. The tumor promotion activity of tobacco smoke is also established [3, 4].

### Relationship between dose–response of each carcinogenic step and clinical outcome

The simplest model to estimate the clinical consequence of a carcinogenic factor, or dose–response relationship between the factor such as tobacco smoking and cancer mortality, is linear-no-threshold, while cancer emerges after progressing through a number of steps in the carcinogenic process [5, 6]. When the dose–response relationship between a factor and each carcinogenic step, such as initiation by mutation induction, is linear, the clinical response is also linear at low doses, called “linearized multistage” [7]. This holds true regardless of whether the factor affects a single or a number of carcinogenic steps. However, this prediction is not applicable at high doses. When the factor affects many carcinogenic steps such as mutation induction and cell growth, the clinical dose–response relationship is more than linear, or a steeper increase in cancer incidence with the increment in dose occurs, even when the dose–response relationship for each step is linear. It is indeed a case of the factor “aging”, since cancer mortality generally increases almost logarithmically with increasing age.

In addition to the traditional Armitage-Doll multistage model, there are many mathematical models to explain the relationship between cancer incidence and age [8–13]. For smoking and smoking-related cancers such as lung cancer, approximately linear dose–response relationships have been observed in a number of epidemiological studies when the dose was represented by the number of cigarettes per day [14–19]. When the dose was represented by the duration of the smoking habit, a greater than linear increment in incidence or mortality was reported [20–22]. Because a life-long smoking habit can be considered as a very high dose of carcinogenic factor, it is possible that the duration of the smoking habit affects several steps of carcinogenesis in a linear relationship, but causes a higher incidence of cancer after long-term smoking. Note that, if a factor affects only one step in a linear dose–response relationship rather than multiple steps described above, the incidence of cancer at a high dose is dependent on which step is affected and the mechanism of cancer induction, such that both a steep increment or a plateau are possible.

### Smoking as an aging accelerator

Naturally, cancer occurs during aging because activation of steps in the carcinogenic process, such as mutation induction and cell growth, increase during aging. Tobacco smoking exhibits a tumor promoting effect as well as induction of mutation, thus smoking induces a number of steps in carcinogenesis. Therefore, when smokers stop smoking, the relative risk of developing cancer gradually decreases compared with the risk in continuing smokers, but it does not fall to the level of nonsmokers, since past smoking continues to have an effect even after long-time smoking cessation. Most studies have demonstrated that the risk in former smokers is far lower than in continuing smokers but remains higher than that in nonsmokers [15, 20–24]. There are several reports concerning cancer risk prediction, from simple approximation by regression equation to rather complicated approaches, including validation of several mathematical models [25–29]. If smoking affects all carcinogenic steps in the same proportion to that of aging, the effects of smoking can be simplified as an additional aging effect, and risk prediction in continuing and former smokers may be possible regardless of the cancer model. I examined such a simple explanation of smoking effects using data reported by Peto et al. [20].

**Fig. 1:**
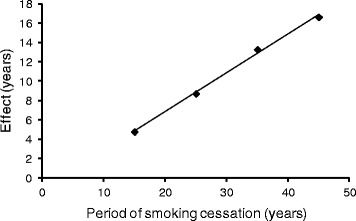
Estimation of the aging effects of smoking. Prolonged smoking cessation resulted in a decreased relative risk of lung cancer mortality, as exhibited by the “delayed effect on aging” (Table 1). The relationship between the period of smoking cessation and the effect was plotted. Linear regression yielded the equation *y* = 0.401*x* − 1.175. The coefficient of the aging effect of smoking was 0.401/(1-0.401) = 0.669. This means that smokers become old 1.669 times faster than nonsmokers. The lag time between smoking and death was 1.175/0.401 = 2.93 years

**Fig. 2:**
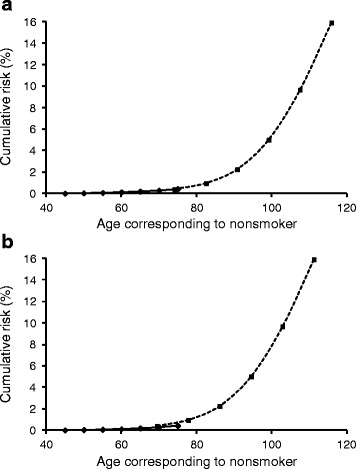
Cumulative lung cancer mortality risk of a nonsmoker and continuing smoker. Data of risk were from Doll et al [20]. The continuing smoker’s age corresponding to that of a nonsmoker was calculated from the coefficient of the aging effect and the lag time (Fig. 1), and age at starting smoking. **a** Smoking started at age 11; **b** Smoking started at age 18. Solid line, nonsmoker; dashed line, continuing smoker. It should be noted that these figures demonstrate the smoking effect as additional aging effect during the smoking period and do not predict the risk in a very old person. It is known that cancer risk in the very old is often lower than that predicted by various cancer models. The sextic regression equation obtained from the risk in a nonsmoker and continuing smoker starting at age 18 together was: *y* = − 5.096171 × 10^− 10^ *x* ^6^ + 1.983542 × 10^− 7^ *x* ^5^ − 3.006177 × 10^− 5^ *x* ^4^ + 2.309032 × 10^− 3^ *x* ^3^ − 0.0953864*x* ^2^ + 2.014889*x* − 17.00096

**Fig. 3:**
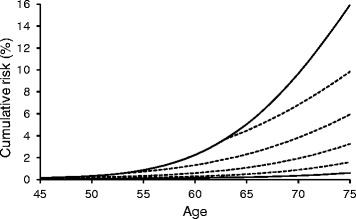
Effect of smoking cessation at various ages on the cumulative lung cancer mortality risk. A single polynomial regression equation (Fig. 2) and age corresponding to a nonsmoker was used. The age of starting smoking was assumed to be 18. The upper and lower solid lines are the risks in continuing smokers and nonsmokers, respectively. The dashed lines are the risks in former smokers who stopped at age 60, 50, 40, and 30 years, from the higher to lower lines

In considering lung cancer death, probability is assumed to be determined by aging level *x* + *a*(*s* − *l*), where *x, s, l,* and *a* are age, smoking period, lag time, and coefficient of aging effect by smoking. The parameters *a* and *l* were estimated from the former smoker’s age corresponding to that of a continuing smoker (Table 1 and Fig. 1). When the risk of nonsmokers and smokers were approximated by a single polynomial equation, the highest correlation was observed if smoking started at 11 years old (Fig. 2). This estimation suggested that smoking effects would be stronger at younger ages, possibly because the smoke would induce mutations at relatively higher levels than promotion of cell growth, compared with older age. A good correlation was still observed by assuming a higher starting age, and thus the age-specific cumulative risks of former smokers were calculated using the equation and assuming a starting age of 18 years (Fig. 3). This risk estimation accorded well with that estimated from the relative risk of the period of smoking cessation [20]. Hence, the effects of the smoking habit may be roughly approximated as an aging effect. Although the data used in this study were from case–control studies combined with national statistics [20], the relative risk of former smokers according to the period of smoking cessation in a variety of large cohort studies in the United States [24] was similar to that of the case–control studies.

**Table 1:** Age and cumulative lung cancer mortality of continuing and former smokers

Age (age at cessation)	Mortality (%)^a^	Age corresponding to smoker^b^
*Continuing smoker*		
50	0.40	
55	0.95	
60	2.25	
65	5.00	
70	9.65	
75	15.9	
*Former smoker*		
75 (60)	9.9	70.22
75 (50)	6.0	66.27
75 (40)	3.0	61.72
75 (30)	1.7	58.37

^a^Data from Doll et al. [20]

^b^The corresponding continuing smoker’s age at which cumulative lung cancer mortality risk is equal to the risk of that in former smokers at age 75 was calculated from the quintic equation^c^ which takes account of the risk in continuing smokers. It is a simple technique and not dependent on the cancer model. The difference between age 75 and the corresponding age is considered to be “delayed effect on aging” by cessation of smoking (Fig. 1)

^c^ *y* = − 1.333333 × 10^− 6^ *x* ^5^ + 3.833333 × 10^− 4^ *x* ^4^ − 0.04306667*x* ^3^ + 2.381417*x* ^2^ − 65.0675*x* + 704.4

A report which analyzed three cohort studies in Japanese subjects suggested that the risk of lung cancer in former smokers decreased to the level in nonsmokers after a long period of smoking cessation [30]. The reason is unclear, but the relative risk of lung cancer in Japanese subjects was lower than that in Caucasians [31, 32], and the detection power was also lower. In addition, the scale of the study was smaller than the cohort studies in Caucasians, thus it is possible that a small difference in risk between long-term quitters and nonsmokers could not be detected.

### Risk estimation - lesson from the smoking effect

When dose is represented by the number of cigarettes per day rather than the duration of the smoking period, approximately linear dose–response relationships are observed in most studies [14–19]. This is in contrast to the effect of smoking duration or aging. From the observation, it is possible that a dose–response relationship between smoking strength and each carcinogenic step, such as initiation and promotion of cancer, is less than linear, or the effects become saturated by a large amount of daily smoking. Using data of large epidemiological studies, the British Doctor Study, the American Cancer Society’s Cancer Prevention Study I and II, the Nurses Health Study and the Health Professionals Follow-Up Study in a two-stage clonal expansion model, the promotion rate powers were estimated to be around 0.5 [28, 29]. This means that the effects of smoking per dose are relatively stronger at lower doses, i.e., second-hand smoking or light smoking. The model simulation provides a different concept from that which suggests there is a threshold for indirect carcinogens. However, it is known that the effects of air pollution and second-hand smoking on cardiovascular mortality per exposure dose are higher than that of active smoking, which has an apparently higher exposure dose [16, 33]. Adverse effects on the cardiovascular system such as oxidative stress, vascular inflammation and dysfunction, activation of the hemostatic system, and disturbance of autonomic nervous system [34, 35], are suggested to be relatively stronger at low doses. In addition, the relative effect of second-hand smoking on lung cancer induction may also be higher than that of active smoking [16, 36]. Therefore, the effects of smoking, such as cancer promotion at low doses may be stronger per dose than at high doses. This case clearly demonstrates the need for careful examination of linear-no-threshold extrapolation as the concept of the “safe side” of risk estimation.

## Conclusion

Smoking is an additional burden on aging and death. It is simple and useful approach to estimate lung cancer risk from duration of smoking habit. Consideration of the dose–response of each effect is important to evaluate the risk arising from each carcinogenic factor including tobacco smoke.
